# Supplementary material for: Perforator versus Non-Perforator Flap-Based Vulvoperineal Reconstruction—A Systematic Review and Meta-Analysis
Source: Cancers (Basel). 2024 Jun 13;16(12):2213. doi: 10.3390/cancers16122213 (PMC11202299; doi:10.3390/cancers16122213)
Supplement: Supplementary file 1 [file cancers-16-02213-s001.zip › cancers-3025986-supplementary.pdf]

Appendix S1: Search strategies

Embase.com

(20220928; 1870 hits)

((('female genital tract tumor'/de OR 'female genital tract cancer'/de OR 'vagina tumor'/exp OR 'vulva tumor'/exp OR 'colorectal tumor'/de OR 'colorectal cancer'/de OR 'anorectal tumor'/exp OR 'pelvis tumor'/exp OR 'perineum tumor'/de OR 'perineum cancer'/de OR 'perineum carcinoma'/de OR 'giant condyloma acuminatum'/de OR (('neoplasm'/exp OR 'cancer surgery'/de OR 'surgical oncology'/de OR 'adenomectomy'/de) AND ('vagina'/exp OR 'perineum'/exp OR 'perineum injury'/de OR 'rectum'/exp OR 'anus'/exp)) OR (((gynecologic* OR gynaecologic* OR pelvis OR pelvic OR 'female genital*' OR vagina OR vagine OR vaginal OR vulva OR vulvar OR perineum OR perineal OR abdominoperineal OR vulvoperineal OR rectum OR rectal OR recti OR mesorectum OR mesorectal OR colorectal OR rectovaginal OR anus OR anal OR anorectal OR anovaginal OR perivulvar OR perirectal OR perianal OR rectosigmoid OR 'lower sigmoid' OR 'lower abdomen' OR Buschke-Lowenstein OR Buschke-Loewenstein) NEAR/3 (cancer* OR neoplas* OR dysplas* OR tumor* OR tumour* OR carcinoma* OR adenocarcinoma OR adenoma* OR carcinogenesis OR malignan* OR melanoma OR polyp* OR pseudopolyp* OR papilloma*)) OR 'giant condyloma'):ab,ti)

AND

('gynecologic surgery'/de OR 'vagina surgery'/de OR 'vaginectomy'/exp OR 'vagina reconstruction'/de OR 'vulvectomy'/de OR 'vulva surgery'/de OR 'perineal surgery'/de OR 'perineal reconstruction'/de OR 'rectum surgery'/de OR 'rectum resection'/exp OR 'anus surgery'/de OR 'anoplasty'/de OR 'abdominoperineal excision'/de OR 'colectomy'/de OR 'sigmoidectomy'/de OR 'reconstructive surgery'/de OR 'plastic surgery'/de OR 'microvascular surgery'/exp OR 'pelvis surgery'/de OR 'pelvis exenteration'/de OR 'pelvis lymphadenectomy'/de OR 'pelvic floor reconstructive surgery'/de OR 'pelvic floor reconstruction'/de OR 'pelvic floor repair'/de OR (((gynecologic* OR gynaecologic* OR pelvis OR pelvic OR 'female genital*' OR vagina OR vagine OR vaginal OR vulva OR vulvar OR perineum OR perineal OR abdominoperineal OR vulvoperineal OR rectum OR rectal OR mesorectum OR mesorectal OR colorectal OR rectovaginal OR anus OR anal OR anorectal OR anovaginal OR perivulvar OR perirectal OR perianal OR rectosigmoid OR 'lower sigmoid' OR 'lower abdomen' OR hindquarter OR local) AND (surgery OR surgical OR repair* OR operation* OR operative OR excis* OR resect* OR amputation OR exstirpation OR extirpation OR exenteration OR lymphadenectom*)) OR colpoplast* OR vaginoplast* OR colpectom* OR colpocleisis OR vaginectom* OR vulvectom* OR anoplast* OR proctectom* OR symphysiotom* OR hemipelvectom* OR colectom* OR sigmoidectom* OR neocolpopoiesis OR 'miles operation' OR 'Brunschwig operation' OR reconstruct* OR 'plastic surg*' OR 'microvascular surg*' OR 'surgical palliation'):ab,ti))

OR

(('female genital tract tumor'/de OR 'female genital tract cancer'/de OR 'vagina tumor'/exp OR 'vulva tumor'/exp OR 'colorectal tumor'/de OR 'colorectal cancer'/de OR 'anorectal tumor'/exp OR 'pelvis tumor'/exp OR 'perineum tumor'/de OR 'perineum cancer'/de OR 'perineum carcinoma'/de OR 'giant condyloma acuminatum'/de OR (('neoplasm'/exp OR 'cancer surgery'/de OR 'surgical oncology'/de OR 'adenomectomy'/de OR (tumor* OR tumour* OR carcinoma* OR adenocarcinoma OR adenoma* OR carcinogenesis OR melanoma OR polyp* OR pseudopolyp* OR papilloma*):ab,ti) AND ('vagina'/exp OR 'perineum'/exp OR 'perineum injury'/de OR 'rectum'/exp OR 'anus'/exp OR (gynecologic* OR gynaecologic* OR pelvis OR pelvic OR 'female genital*' OR vagina OR vagine OR vaginal OR vulva OR vulvar OR perineum OR perineal OR abdominoperineal OR vulvoperineal OR rectum OR rectal OR recti OR mesorectum OR mesorectal OR colorectal OR rectovaginal OR anus OR anal OR anorectal OR anovaginal OR perivulvar OR perirectal OR perianal OR rectosigmoid OR 'lower sigmoid' OR 'lower abdomen' OR

Buschke-Lowenstein OR Buschke-Loewenstein):ab,ti)) OR ('giant condyloma' OR oncolog* OR cancer OR neoplas* OR dysplas* OR malignan*):ab,ti)

AND

('gynecologic surgery'/de OR 'vagina surgery'/de OR 'vaginectomy'/exp OR 'vagina reconstruction'/de OR 'vulvectomy'/de OR 'vulva surgery'/de OR 'perineal surgery'/de OR 'perineal reconstruction'/de OR 'rectum surgery'/de OR 'rectum resection'/exp OR 'anus surgery'/de OR 'anoplasty'/de OR 'abdominoperineal excision'/de OR 'colectomy'/de OR 'sigmoidectomy'/de OR 'pelvis surgery'/de OR 'pelvis exenteration'/de OR 'pelvis lymphadenectomy'/de OR 'pelvic floor reconstructive surgery'/de OR 'pelvic floor reconstruction'/de OR 'pelvic floor repair'/de OR (('reconstructive surgery'/de OR 'plastic surgery'/de OR 'microvascular surgery'/exp) AND ('vagina'/exp OR 'perineum'/exp OR 'perineum injury'/de OR 'rectum'/exp OR 'anus'/exp)) OR (((gynecologic* OR gynaecologic* OR pelvis OR pelvic OR 'female genital*' OR vagina OR vagine OR vaginal OR vulva OR vulvar OR perineum OR perineal OR abdominoperineal OR vulvoperineal OR rectum OR rectal OR mesorectum OR mesorectal OR colorectal OR rectovaginal OR anus OR anal OR anorectal OR anovaginal OR perivulvar OR perirectal OR perianal OR rectosigmoid OR 'lower sigmoid' OR 'lower abdomen' OR hindquarter) NEAR/5 (surgery OR surgical OR reconstruct* OR repair* OR operation* OR operative OR excis* OR resect* OR amputation OR exstirpation OR extirpation OR exenteration OR lymphadenectom*)) OR colpoplast* OR vaginoplast* OR colpectom* OR colpocleisis OR vaginectom* OR vulvectom* OR anoplast* OR proctectom* OR symphysiotom* OR hemipelvectom* OR colectom* OR sigmoidectom* OR neocolpopoiesis OR 'miles operation' OR 'Brunschwig operation'):ab,ti)))

AND

('surgical flaps'/exp OR 'tissue flap'/de OR 'adipofascial flap'/de OR 'anterolateral thigh flap'/de OR 'chimeric flap'/de OR 'deep inferior epigastric perforator flap'/de OR 'fasciocutaneous flap'/de OR 'gracilis flap'/de OR 'inferior gluteal artery perforator flap'/de OR 'inguinal flap'/de OR 'island flap'/de OR 'latissimus dorsi flap'/de OR 'muscle flap'/de OR 'myocutaneous flap'/de OR 'perforator flap'/de OR 'skin flap'/exp OR 'transverse rectus abdominis musculocutaneous flap'/de OR 'vertical rectus abdominis musculocutaneous flap'/de OR 'venous flap'/de OR 'omental flap'/de OR 'v y plasty'/de OR 'skin graft'/de OR 'full thickness skin graft'/de OR (((surgical OR free OR tissue OR adipofascial OR adipo-fascial OR 'anterolateral thigh' OR 'antero-lateral thigh' OR ALT OR 'medial thigh' OR 'posterior thigh' OR pudendal-thigh OR chimeric OR fasciocutaneous OR fascio-cutaneous OR gracilis OR IGAP OR I-GAP OR inguinal OR groin OR island OR muscle OR myocutaneous OR myo-cutaneous OR muscular-cutaneous OR musculocutaneous OR musculo-cutaneous OR perforator OR skin OR cutaneous OR dermal OR pedicle* OR bipedicle* OR transposition* OR Singapore OR VRAM OR gluteal OR gluteus OR omentum OR omental OR peritoneum OR peritoneal OR V-Y OR fillet OR local OR advancement OR 'fasciae latae' OR Martius) NEAR/3 (flap OR flaps OR graft OR grafts)) OR 'free inguinal graft' OR 'skin pedicle' OR 'flap repair' OR 'flap reconstruction*' OR 'V-Y plasty'):ab,ti)

NOT

(('animal'/de OR 'animal experiment'/exp OR 'nonhuman'/de) NOT ('human'/exp OR 'human experiment'/de))

NOT

[conference abstract]/lim

Medline (Ovid)

(20220628; Ovid MEDLINE(R) ALL 1946 to September 27, 2022; 1598 hits)

((("genital neoplasms, female"/ OR exp vaginal neoplasms/ OR exp vulvar neoplasms/ OR colorectal neoplasms/ OR exp rectal neoplasms/ OR sigmoid neoplasms/ OR pelvic neoplasms/ OR (exp neoplasms/ AND (exp vagina/ OR exp vulva/ OR perineum/ OR rectum/ OR anal canal/)) OR (((gynecologic* OR gynaecologic* OR pelvis OR pelvic OR female genital* OR vagina OR vagine OR vaginal OR vulva OR vulvar OR perineum OR perineal OR abdominoperineal OR vulvoperineal OR rectum OR rectal OR recti OR

mesorectum OR mesorectal OR colorectal OR rectovaginal OR anus OR anal OR anorectal OR anovaginal OR perivulvar OR perirectal OR perianal OR rectosigmoid OR lower sigmoid OR lower abdomen OR Buschke-Lowenstein OR Buschke-Loewenstein) ADJ3 (cancer* OR neoplas* OR dysplas* OR tumor* OR tumour* OR carcinoma* OR adenocarcinoma OR adenoma* OR carcinogenesis OR malignan* OR melanoma OR polyp* OR pseudopolyp* OR papilloma*)) OR giant condyloma).ab,ti.)

AND

(gynecologic surgical procedures/ OR exp vagina/su OR exp vulva/su OR exp perineum/su OR exp rectum/su OR anal canal/su OR vulvectomy/ OR reconstructive surgical procedures/ OR surgery, plastic/ OR exp pelvis/su OR (((gynecologic* OR gynaecologic* OR pelvis OR pelvic OR female genital* OR vagina OR vagine OR vaginal OR vulva OR vulvar OR perineum OR perineal OR abdominoperineal OR vulvoperineal OR rectum OR rectal OR mesorectum OR mesorectal OR colorectal OR rectovaginal OR anus OR anal OR anorectal OR anovaginal OR perivulvar OR perirectal OR perianal OR rectosigmoid OR lower sigmoid OR lower abdomen OR hindquarter OR local) AND (surgery OR surgical OR repair* OR operation* OR operative OR excis* OR resect* OR amputation OR exstirpation OR extirpation OR exenteration OR lymphadenectom*)) OR colpoplast* OR vaginoplast* OR colpectom* OR colpocleisis OR vaginectom* OR vulvectom* OR anoplast* OR proctectom* OR symphysiotom* OR hemipelvectom* OR colectom* OR sigmoidectom* OR neocolpopoiesis OR miles operation OR Brunschwig operation OR reconstruct* OR plastic surg* OR microvascular surg* OR surgical palliation).ab,ti.))

OR

(("genital neoplasms, female"/ OR exp vaginal neoplasms/ OR exp vulvar neoplasms/ OR colorectal neoplasms/ OR exp rectal neoplasms/ OR sigmoid neoplasms/ OR pelvic neoplasms/ OR ((exp neoplasms/ OR (tumor* OR tumour* OR carcinoma* OR adenocarcinoma OR adenoma* OR carcinogenesis OR melanoma OR polyp* OR pseudopolyp* OR papilloma*).ab,ti.) AND (exp vagina/ OR exp vulva/ OR perineum/ OR rectum/ OR anal canal/ OR (gynecologic* OR gynaecologic* OR pelvis OR pelvic OR female genital* OR vagina OR vagine OR vaginal OR vulva OR vulvar OR perineum OR perineal OR abdominoperineal OR vulvoperineal OR rectum OR rectal OR recti OR mesorectum OR mesorectal OR colorectal OR rectovaginal OR anus OR anal OR anorectal OR anovaginal OR perivulvar OR perirectal OR perianal OR rectosigmoid OR lower sigmoid OR lower abdomen OR Buschke-Lowenstein OR Buschke-Loewenstein).ab,ti.)) OR (giant condyloma OR oncolog* OR cancer OR neoplas* OR dysplas* OR malignan*).ab,ti.)

AND

(gynecologic surgical procedures/ OR exp vagina/su OR exp vulva/su OR exp perineum/su OR exp rectum/su OR anal canal/su OR vulvectomy/ OR exp pelvis/su OR ((reconstructive surgical procedures/ OR surgery, plastic/) AND (exp vagina/ OR exp vulva/ OR perineum/ OR rectum/ OR anal canal/)) OR (((gynecologic* OR gynaecologic* OR pelvis OR pelvic OR female genital* OR vagina OR vagine OR vaginal OR vulva OR vulvar OR perineum OR perineal OR abdominoperineal OR vulvoperineal OR rectum OR rectal OR mesorectum OR mesorectal OR colorectal OR rectovaginal OR anus OR anal OR anorectal OR anovaginal OR perivulvar OR perirectal OR perianal OR rectosigmoid OR lower sigmoid OR lower abdomen OR hindquarter) ADJ5 (surgery OR surgical OR reconstruct* OR repair* OR operation* OR operative OR excis* OR resect* OR amputation OR exstirpation OR extirpation OR exenteration OR lymphadenectom*)) OR colpoplast* OR vaginoplast* OR colpectom* OR colpocleisis OR vaginectom* OR vulvectom* OR anoplast* OR proctectom* OR symphysiotom* OR hemipelvectom* OR colectom* OR sigmoidectom* OR neocolpopoiesis OR miles operation OR Brunschwig operation).ab,ti.)))

AND

(exp surgical flaps/ OR (((surgical OR free OR tissue OR adipofascial OR adipo-fascial OR anterolateral thigh OR antero-lateral thigh OR ALT OR medial thigh OR posterior thigh OR pudendal-thigh OR chimeric OR fasciocutaneous OR fascio-cutaneous OR gracilis OR IGAP OR I-GAP OR inguinal OR groin OR island OR muscle OR myocutaneous OR myo-cutaneous OR muscular-cutaneous OR musculocutaneous OR musculo-

cutaneous OR perforator OR skin OR cutaneous OR dermal OR pedicle* OR bipedicle* OR transposition* OR Singapore OR VRAM OR gluteal OR gluteus OR omentum OR omental OR peritoneum OR peritoneal OR V-Y OR fillet OR local OR advancement OR fasciae latae OR Martius) ADJ3 (flap OR flaps OR graft OR grafts)) OR free inguinal graft OR skin pedicle OR flap repair OR flap reconstruction* OR V-Y plasty).ab,ti.)

NOT (exp animals/ NOT humans/)

Web of Science Core Collection

(20220028; Web of Science Core Collection, Editions = A&HCI , BKCI-SSH , BKCI-S , CCR-EXPANDED , ESCI , IC , CPCI-SSH , CPCI-S , SCI-EXPANDED , SSCI; 1242 hits)

TS=((((((((gynecologic* OR gynaecologic* OR pelvis OR pelvic OR "female genital*" OR vagina OR vagine OR vaginal OR vulva OR vulvar OR perineum OR perineal OR abdominoperineal OR vulvoperineal OR rectum OR rectal OR recti OR mesorectum OR mesorectal OR colorectal OR rectovaginal OR anus OR anal OR anorectal OR anovaginal OR perivulvar OR perirectal OR perianal OR rectosigmoid OR "lower sigmoid" OR "lower abdomen" OR Buschke-Lowenstein OR Buschke-Loewenstein) NEAR/2 (cancer* OR neoplas* OR dysplas* OR tumor* OR tumour* OR carcinoma* OR adenocarcinoma OR adenoma* OR carcinogenesis OR malignan* OR melanoma OR polyp* OR pseudopolyp* OR papilloma*)) OR "giant condyloma")))

AND

((((gynecologic* OR gynaecologic* OR pelvis OR pelvic OR "female genital*" OR vagina OR vagine OR vaginal OR vulva OR vulvar OR perineum OR perineal OR abdominoperineal OR vulvoperineal OR rectum OR rectal OR mesorectum OR mesorectal OR colorectal OR rectovaginal OR anus OR anal OR anorectal OR anovaginal OR perivulvar OR perirectal OR perianal OR rectosigmoid OR "lower sigmoid" OR "lower abdomen" OR hindquarter OR local) AND (surgery OR surgical OR repair* OR operation* OR operative OR excis* OR resect* OR amputation OR exstirpation OR extirpation OR exenteration OR lymphadenectom*)) OR colpoplast* OR vaginoplast* OR colpectom* OR colpocleisis OR vaginectom* OR vulvectom* OR anoplast* OR proctectom* OR symphysiotom* OR hemipelvectom* OR colectom* OR sigmoidectom* OR neocolpopoiesis OR "miles operation" OR "Brunschwig operation" OR reconstruct* OR "plastic surg*" OR "microvascular surg*" OR "surgical palliation")))

OR

(((((tumor* OR tumour* OR carcinoma* OR adenocarcinoma OR adenoma* OR carcinogenesis OR melanoma OR polyp* OR pseudopolyp* OR papilloma*) AND (gynecologic* OR gynaecologic* OR pelvis OR pelvic OR "female genital*" OR vagina OR vagine OR vaginal OR vulva OR vulvar OR perineum OR perineal OR abdominoperineal OR vulvoperineal OR rectum OR rectal OR recti OR mesorectum OR mesorectal OR colorectal OR rectovaginal OR anus OR anal OR anorectal OR anovaginal OR perivulvar OR perirectal OR perianal OR rectosigmoid OR "lower sigmoid" OR "lower abdomen" OR Buschke-Lowenstein OR Buschke-Loewenstein)) OR "giant condyloma" OR oncolog* OR cancer OR neoplas* OR dysplas* OR malignan*))

AND

((((gynecologic* OR gynaecologic* OR pelvis OR pelvic OR "female genital*" OR vagina OR vagine OR vaginal OR vulva OR vulvar OR perineum OR perineal OR abdominoperineal OR vulvoperineal OR rectum OR rectal OR mesorectum OR mesorectal OR colorectal OR rectovaginal OR anus OR anal OR anorectal OR anovaginal OR perivulvar OR perirectal OR perianal OR rectosigmoid OR "lower sigmoid" OR "lower abdomen" OR hindquarter) NEAR/5 (surgery OR surgical OR reconstruct* OR repair* OR operation* OR operative OR excis* OR resect* OR amputation OR exstirpation OR extirpation OR exenteration OR lymphadenectom*)) OR colpoplast* OR vaginoplast* OR colpectom* OR colpocleisis OR vaginectom* OR vulvectom* OR anoplast* OR proctectom* OR symphysiotom* OR hemipelvectom* OR colectom* OR sigmoidectom* OR neocolpopoiesis OR "miles operation" OR "Brunschwig operation"))))

AND

(((surgical OR free OR tissue OR adipofascial OR adipo-fascial OR "anterolateral thigh" OR "antero-lateral thigh" OR ALT OR "medial thigh" OR "posterior thigh" OR pudendal-thigh OR chimeric OR fasciocutaneous OR fascio-cutaneous OR gracilis OR IGAP OR I-GAP OR inguinal OR groin OR island OR muscle OR myocutaneous OR myo-cutaneous OR muscular-cutaneous OR musculocutaneous OR musculo-cutaneous OR perforator OR skin OR cutaneous OR dermal OR pedicle* OR bipedicle* OR transposition* OR Singapore OR VRAM OR gluteal OR gluteus OR omentum OR omental OR peritoneum OR peritoneal OR V-Y OR fillet OR local OR advancement OR "fasciae latae" OR Martius) NEAR/2 (flap OR flaps OR graft OR grafts)) OR "free inguinal graft" OR "skin pedicle" OR "flap repair" OR "flap reconstruction*" OR "V-Y plasty"))

NOT DT=(Meeting Abstract)

Search narrative

The first two search concepts ("pelvic cancer" and "pelvic surgery") were duplicated and worked out in two different ways which, in turn, were combined with OR. In the first way, general cancer terms like "oncolog*" or "cancer" were included without restriction with an anatomical term of the pelvis. In the second way, general surgery terms such as "reconstruct*", "plastic surgery" or "microvascular surgery" were included without restriction with an anatomical term of the pelvis. The reason for this was that anatomical terms of the pelvis could be explicitly named by the authors only in connection with either cancer or surgery but not necessarily with both.

Also, the proximity operator was replaced by AND in those "pelvic cancer" and "pelvic surgery" concepts that were not restrained by anatomical terms. For the "pelvic cancer" concept, this replacement with AND also involved the following remodeling: The nested ANDed brackets *((anatomy terms) AND (cancer terms))* with subject headings and text words were fused.

Table S1. Statistics 2023-Complications after Perforator vs non-Perforator Flap Transplanation.

Included Studies

			**# Patients**					
Study	Country	N	PF	NPF	both	Age	BMI	Disease
Abdulrahman 2022 [22]	United Kingdom	13	0	13	0	65	30 [20 to 40]	Squamous cell carcinoma
Chen 2022 [23]	China	30	0	30	0	58	25 [20 to 32]	
Elia 2022 [59]	Taiwan	33	29	4	0	60 (16)	23	Squamous cell carcinoma, Vulvar intraepithelial neoplasia, Malignant melanoma, Paget's disease, Sarcoma, Dermatofibrosarcoma, Bartholini gland tumor, Verrucous carcinoma, Condyloma
Muallem 2022 [24]	Germany	126	0	126	0	70		Keratinizing squamous carcinoma, Non keratinizing squamous carcinoma, Basaloid carcinoma, Adenocarcinoma
Shin 2022 [51]	Korea	16	16	0	0	Na		Squamous cell carcinoma, Malignant melanoma, Paget's disease
Zhang 2022 [61]	China	26	0	24	2	55		Squamous cell carcinoma, Dermatofibrosarcoma, Adenocarcinoma, Sebaceous gland carcinoma
Aksan 2021 [25]	Turkey	18	0	18	0	62 (13)	29 [24 to 39]	Squamous cell carcinoma, High-grade squamouse intraepithelial lesion, Paget's disease, Malignant Melanoma
Giannini 2021 [26]	United Kingdom	23	0	23	0	60 (16)	28	Squamous cell carcinoma
Tock 2019 [27]	France	61	0	61	0	Na		Squamous cell carcinoma, Not specified
Fin 2018 [28]	Italy	30	0	30	0	75		Squamous cell carcinoma

Hand 2018 [29]	USA	27	0	27	0	69	29 [18 to 51]	Squamous cell carcinoma, Paget's disease, Dermatofibrosarcoma, Endometrial adenocarcinoma, Bartholini Cyst, Hidradenitis suppurativa, Chronic fistula, Vulvar intraepithelial neoplasia, Vaginal smooth muscle tumor, Lymphangiectasia
Hellinga 2018 [9]	Netherlands	59	0	59	0	Na		Vulvar cancer, Lichen sclerosus, Morbus Zoon
Confalonieri 2017 [10]	Italy	23 4	0	234	0	69		Squamous cell carcinoma, Carcinoma, Malignant melanoma, Paget's disease, Vulvar intraepithelial neoplasia, Not specified
Lange 2017 [20]	Netherlands	75	0	75	0	72 (14)	27	Squamous cell carcinoma, Vulvar intraepithelial neoplasia, Paget's disease, Basal cell carcinoma
Chang 2016 [21]	Taiwan	12	12	0	0	60	25 [19 to 28]	Squamous cell carcinoma, Malignant melanoma, Vulvar intraepithelial neoplasia, Dermatofibrosarcoma
Conri 2016 [30]	France	21	0	21	0	76	28 [18 to 36]	Squamous cell carcinoma, Paget's disease, Malignant melanoma
Gentileschi 2017 [52]	Italy	15	15	0	0	63	26	Squamous cell carcinoma, Sarcomatoid carcinoma, Endometrial Cancer
Herraiz Roda 2016 [31]	Spain	9	0	9	0	79	29.6 [23 to 36]	Squamous cell carcinoma, Paget's disease
Huang 2015 [53]	Taiwan	16	16	0	0	56 (18)	24 [19 to 29]	Squamous cell carcinoma, Paget's disease, Vulvar intraepithelial neoplasia, Malignant Melanoma, Bartholini gland carcinoma, Dermatofibrosarcoma
Kim 2015 [32]	Korea	23	0	23	0	52	27 [22 to 33]	Vulva Cancer, Extramammaey Paget's Disease, Vulva dysplasia, Vulva and Vagina Cancer
Negosanti 2015 [62]	Italy	22	5	17	0	Na		Squamous cell carcinoma, Paget's disease, Lichen sclerosus, Leiomyosarkom, Malignant melanoma
Nomura 2015 [33]	Japan	19	0	19	0	65		
Zhang 2015 [63]	China	24	22	0	2	50		Squamous cell carcinoma, Neuroectodermal tumor, Sarcoma, Hemangioendotheliom, Malignant melanoma, Bartholini gland tumor
Zhang 2015_2 [64]	China	36	33	1	2	50 (13)	24 [19 to 36]	Squamous cell carcinoma, Malignant melanoma, Sarcoma, Bartholini gland carcinoma, Not specified
Benedetti Panici 2014 [34]	Italy	29	0	29	0	70	25 [22 to 38]	Squamous cell carcinoma, Malignant melanoma, Adenocarcinoma
Tan 2014 [60]	Singapore	39	0	38	1	61		Squamous cell carcinoma, Paget's disease, Dermatofibrosarcoma, Naevus, Vulvar sinus, Necrotizing fasciitis, Adenocarcinoma, Mucinous carcinoma, Sarcoma, Clear cell carcinoma
Argenta 2013 [35]	United Kingdom	59	0	59	0	59	27 [19 to 34]	Vulvar Cancer, Vulvar dysplasia, Hidradenitis suppurativa, Colorectal disease
Kuokkanen 2013 [36]	Finland	22	0	22	0	78		Epidermoid carcinoma, Paget's disease, Malignant melanoma
Lee 2013 [37]	Korea	16	0	16	0	58		Vulva cancer, Extramammaey Paget's Disease, Angiomyxoma
Al-Benna 2012 [54]	Germany	8	8	0	0	56 (6)		vulvar intraepithelial neoplasia, Vulvar Cancer, Paget's disease
Buda 2012 [38]	Italy	22	0	22	0	64	28 [16 to 39]	Vulvar Cancer, Introital stenosis
Misani 2011 [39]	Italy	41	0	41	0	72	30 [25 to 53]	Squamous cell carcinoma
Yun 2010 [55]	Korea	15	15	0	0	56		Vulvar Cancer, Paget's disease, Fistula
Zeng 2011 [66]	China	11	8	0	3	52		Vulvar Cancer
Zhou 2010 [56]	China	10	10	0	0	58		Squamous cell carcinoma, Verrucous carcinoma, Paget's disease, Periurethral carcinoma
Franchelli 2009 [57]	Italy	33	33	0	0	66		Squamous cell carcinoma, Paget's disease, Malignant melanoma, Carcinoma in situ
Staiano 2007 [40]	United Kingdom	46	0	46	0	64		Squamous cell carcinoma, Extramammaey Paget's Disease, Adenocarcinoma, Leiomyosarcoma, Melanoma, Basosquamous carcinoma

Weikel 2006 [42]	Ger-many	12 3	0	123	0	67	Vulvar Cancer
Weikel 2008 [41]	Ger-many	20 7	0	207	0	64	Vulvar carcinoma
Salgarello 2005 [65]	Italy	20	4	13	3	69	Squamous cell carcinoma, Basal cell carcinoma, Paget's disease
Ragoowansi 2004 [58]	United King-dom	40	40	0	0	57	Squamous cell carcinoma, Vulvar intraepithelial neo-plasia, Fistula, Hidradenitis suppurativa
Persichetti 2003 [43]	Italy	26	0	26	0	Na	Epidermoid carcinoma, Paget's disease, Malignant melanoma
Arkoulakis 2002 [44]	USA	20	0	20	0	68	Malignant Melanoma, Paget's disease, Adenocarcinoma, Verrucouse carcinoma, Squamous cell carcinoma
Moschella 1999 [45]	Italy	22	0	22	0	57	Squamous cell carcinoma, Vulvar intraepithelial neo-plasia, Premalignant lesions
Loree 1997 [46]	USA	7	0	7	0	59	Vulvar Cancer, Paget's disease, Enteroperineal fistula, Adenocarcinoma
Niranjan 1996 [47]	United King-dom	8	0	8	0	59	Squamous cell carcinoma, Vulvar intra epithelial neo-plasia, Stenosis
Burke 1994 [48]	USA	17	0	17	0	55	Squamous cell carcinoma, Adenocarcinoma, Malignant melanoma, Cloacogenic carcinoma
Helm 1993 [49]	USA	15	0	15	0	65	Squamous cell carcinoma, Bartholini gland carcinoma, Vulvar intraepithelial neoplasia, Vaginal intraepithe-lial neoplasia
Shepeherd 1990 [50]	United King-dom	16	0	16	0	55	Vulvar Cancer
